# Tackling student neurophobia in neurosciences block with team-based learning

**DOI:** 10.3402/meo.v20.28461

**Published:** 2015-07-30

**Authors:** Khurshid Anwar, Abdul A. Shaikh, Muhammad R. Sajid, Peter Cahusac, Norah A. Alarifi, Ahlam Al Shedoukhy

**Affiliations:** 1 Department of Pathology, College of Medicine, Alfaisal University, Riyadh, Saudi Arabia; 2 Department of Physiology, College of Medicine, Alfaisal University, Riyadh, Saudi Arabia; 3 Department of Pharmacology, College of Medicine, Alfaisal University, Riyadh, Saudi Arabia; 4 Fourth year Medical Student, College of Medicine, Alfaisal University, Riyadh, Saudi Arabia

**Keywords:** team-based learning, neurosciences block, small group teaching, neurophobia

## Abstract

**Introduction:**

Traditionally, neurosciences is perceived as a difficult course in undergraduate medical education with literature suggesting use of the term “Neurophobia” (fear of neurology among medical students). Instructional strategies employed for the teaching of neurosciences in undergraduate curricula traditionally include a combination of lectures, demonstrations, practical classes, problem-based learning and clinico-pathological conferences. Recently, team-based learning (TBL), a student-centered instructional strategy, has increasingly been regarded by many undergraduate medical courses as an effective method to assist student learning.

**Methods:**

In this study, 156 students of year-three neuroscience block were divided into seven male and seven female groups, comprising 11–12 students in each group. TBL was introduced during the 6 weeks of this block, and a total of eight TBL sessions were conducted during this duration. We evaluated the effect of TBL on student learning and correlated it with the student's performance in summative assessment. Moreover, the students’ perceptions regarding the process of TBL was assessed by online survey.

**Results:**

We found that students who attended TBL sessions performed better in the summative examinations as compared to those who did not. Furthermore, students performed better in team activities compared to individual testing, with male students performing better with a more favorable impact on their grades in the summative examination. There was an increase in the number of students achieving higher grades (grade B and above) in this block when compared to the previous block (51.7% vs. 25%). Moreover, the number of students at risk for lower grades (Grade B- and below) decreased in this block when compared to the previous block (30.6% vs. 55%). Students generally elicited a favorable response regarding the TBL process, as well as expressed satisfaction with the content covered and felt that such activities led to improvement in communication and interpersonal skills.

**Conclusion:**

We conclude that implementing TBL strategy increased students’ responsibility for their own learning and helped the students in bridging the gap in their cognitive knowledge to tackle ‘neurophobia’ in a difficult neurosciences block evidenced by their improved performance in the summative assessment.

Neuroscience is an essential course in undergraduate medical education, with an abundance of recommendations for how and what to teach (1). It is also one of the most difficult with literature suggesting use of the term ‘Neurophobia’ among undergraduate medical students (2). Instructional strategies employed for this purpose traditionally include lectures, demonstrations, practical classes; problem-based learning (PBL); and clinico-pathological conferences with other newer strategies including online learning and videos (3). Recently, team-based learning (TBL) as an instructional strategy has gained acceptance in the teaching of undergraduate medical curricula, as it promotes active learning (4–6). TBL is a student-centered, subject specialist-directed instructional strategy, providing students with opportunities to apply their knowledge through a series of activities comprising individual work, team work, immediate feedback, and its application to problem-solving task-based assignments (4). The major benefit of TBL is that it incorporates the effectiveness of small group learning into large group sessions. This combination of small and large group dynamics leads to a high degree of interaction among learners, while tutors retain control over content delivered in the session and its mode of delivery (4–6).

Traditional TBL consists of three stages: 1) advanced student preparation based upon provided session objectives; 2) preparation assessment by i-RAT (individual readiness assurance test) and t-RAT (team readiness assurance test) followed by immediate feedback; and 3) group-based problem solving in the context of a provided clinical scenario (4–6).

We have tried to follow the guidelines suggested by Haidet et al. in reporting the results of our study and the process of TBL (7). The undergraduate curriculum of our university is an integrated hybrid PBL curriculum. We recently introduced TBL methodology in the neuroscience module in year-3 of the curriculum. The aim of this study was to evaluate the effect of TBL on student learning and to correlate it with the student's performance in summative assessment. This module was chosen for this intervention because of the perceived difficulty of the neuroscience subject (2, 3).

The second objective was to evaluate the students’ perceptions regarding the process of TBL. To Our knowledge, this is the first time a TBL strategy was implemented in any of the medical colleges of Saudi Arabia, utilizing state-of-the art teaching equipment, truly representing the first example of a novel teaching system within the region.

## Methods

This project was reviewed and approved by the university institutional review board. The four essential principles of team learning as outlined by Michaelsen: 1) groups must be properly formed and managed; 2) students must be made accountable for their individual and group work; 3) group assignments must promote both learning and team development; and 4) students must have frequent and timely feedback, were followed (7–10). For this study, all the students (*n*=156) of the third year, six-week long, neuroscience block, were divided into seven male and seven female groups comprising 11–12 students in each group.

During this six-week block, a total of eight TBL sessions were conducted, each session lasting for two hours with separate sessions for male and female students. The objectives for the TBL session were posted on Moodle^®^ 1 week before the TBL session. Before the start of i-RAT, faculty members ensured that the students were seated as in an exam arrangement. While in t-RAT the students sat in groups facing each other to maximize team work. i-RATs were marked using Scantrons^TM^ from all students. t-RATs were conducted after completion of the i-RAT, and only group scores submitted by group leaders were used. TBL sessions comprised ten multiple choice questions (MCQs) and were conducted by two faculty members. Students were given 75 s for each MCQ during i-RAT and t-RAT. All MCQs were provided by subject specialists, and consisted of a short clinical vignette, laboratory data, imaging studies, and high-resolution photomicrographs depicting gross and histopathological findings covering that week's objectives. The MCQs were in the form of a timed PowerPoint presentation, which was projected on a SMART Board^TM^.

Answers to the i-RAT and t-RAT questions were marked on Scantrons^TM^ by the students. During the t-RAT, the students worked in predefined groups led by a group leader. It was the job of the group leader to bubble the answer on Scantrons^TM^, after reaching a consensus or show by hands, the answer of the majority if no consensus was reached.

Individual and team activities were followed by providing immediate feedback to students with discussion on all MCQs. In case of disagreement in answers, groups were encouraged to defend their answers by providing logical reasoning. The clinical scenarios in the MCQs were discussed as stage three of TBL. The students were allowed to challenge the quality of materials presented in the clinical scenarios and MCQs. The Scantrons^TM^ sheets were processed on Scantrons^TM^ software and the results were exported to SPSS version 21. The results were also manually verified and adjusted for the number of sessions attended by the students. A 24-item online anonymous questionnaire was also designed using a Likert scale. It was posted on SurveyMonkey for the collection of students’ feedback on the TBL process. An email was sent to all the students asking them to fill the questionnaire.

### Statistical methods

Statistical analyses of data from i-RAT and t-RAT were performed using SPSS statistical software (SPSS, Inc. v. 21, Chicago, IL, USA). We calculated the mean, standard deviation, and standard error of mean with 95% confidence interval for i-RAT and t-RAT scores and compared individual scores with team scores for all questions using scatterplots and bar graphs. Pearson's correlation was used to evaluate the impact of TBL on summative examination scores for all students as well as to calculate gender differences and plotted graphically. For all analyses, *p*<0.05 was considered statistically significant. The survey questionnaire was organised in Excel according to the Likert scale (SD=strongly disagree, D=disagree, N=neutral, A=agree, and SA=strongly agree) and descriptive statistics were calculated.

## Results

### i-RAT and t-RAT

**Fig. 1:**
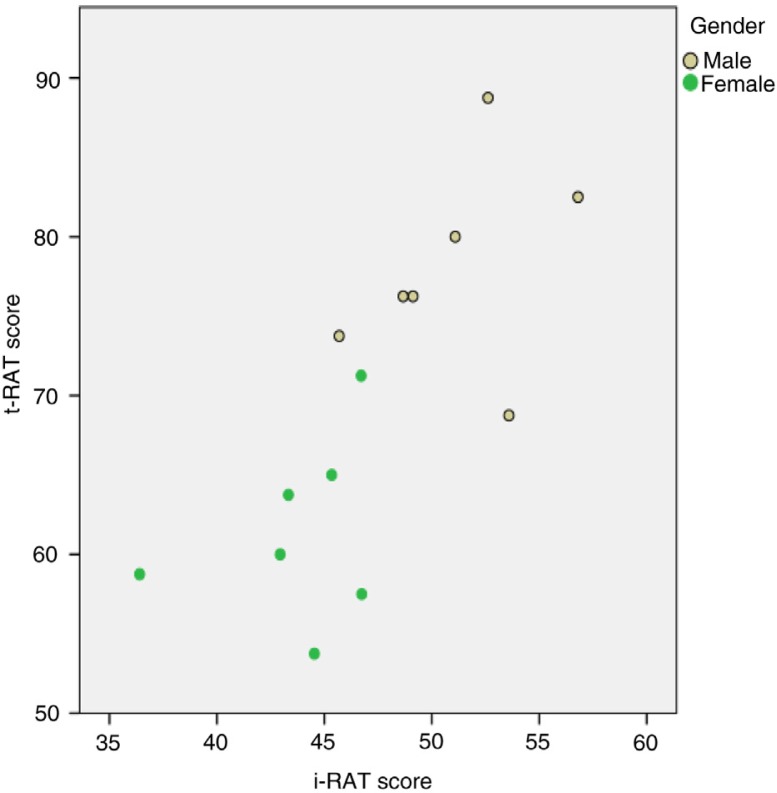
Scatterplot of t-RAT score against i-RAT score. The overall correlation was strong (*r*=0.74, *p*=0.003, *N*=14). The correlations for each gender were weaker and not statistically significant (male: *r*=0.35, *p*=0.44; female: *r*=0.30, *p*=0.51). The combined correlation was overestimated due to heterogeneity of gender subgroups.

All third year medical students (*n*=156) attended the TBL sessions. There were a total of 14 groups (7 males and 7 females), and the statistical analysis for i-RAT for these groups showed that the mean i-RAT scores for all groups was 47% and mean t-RAT score was 70%. Figure 1 shows scatterplot of t-RAT scores against i-RAT scores for all groups. The overall correlation was strong (*r*=0.74, *p*=0.003, and *N*=14).

### Gender differences

**Fig. 2:**
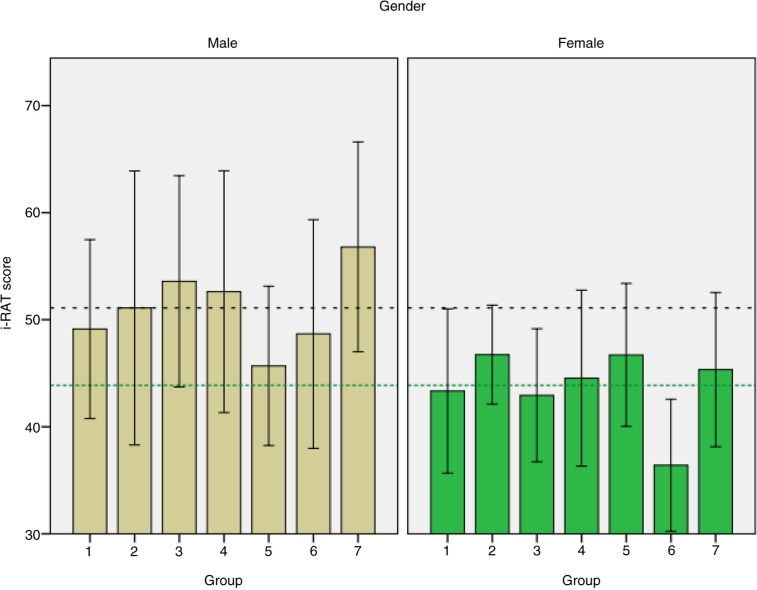
Bar graphs of i-RAT scores from 14 groups split according to gender. The bars show means±95% confidence interval error bars. The overall mean i-RAT for males is indicated by the horizontal black dashed line, and the overall mean for females as the horizontal green dotted line.

The mean i-RAT and t-RAT scores were 51 and 78% for male students and 44 and 61% for female students. This was statistically significant (*p*<0.001) as shown in Figs. 1 and 2. The correlations for each gender were weaker and not statistically significant (male: *r*=0.35, *p*=0.44; female: *r*=0.30, *p*=0.51). The combined correlation was overestimated due to heterogeneity of gender subgroups as shown in Fig. 1.

### Attendance and summative examination

**Fig. 3:**
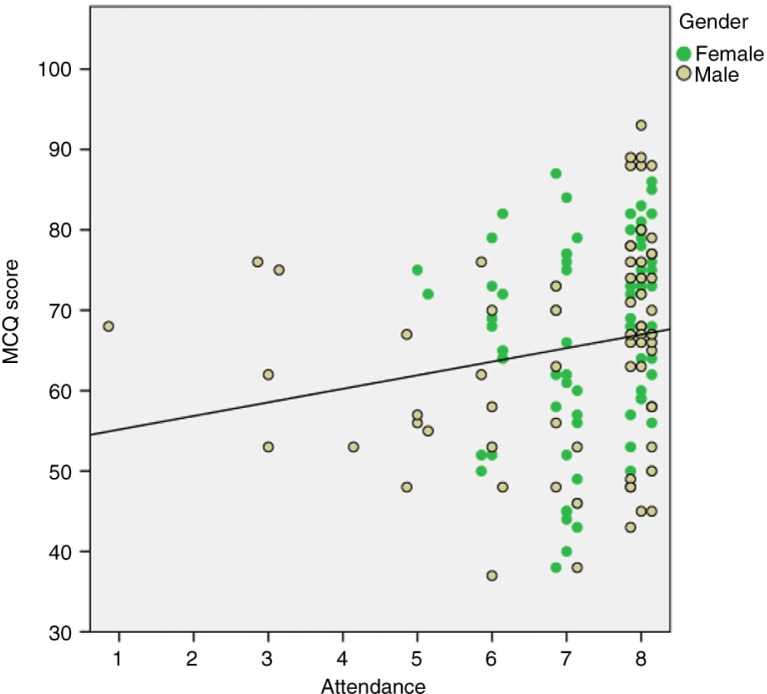
Weak correlation between the students attending the sessions and their summative exam MCQ scores (Spearman's ρ=0.27, *p*=0.001, *N*=143).

**Fig. 4:**
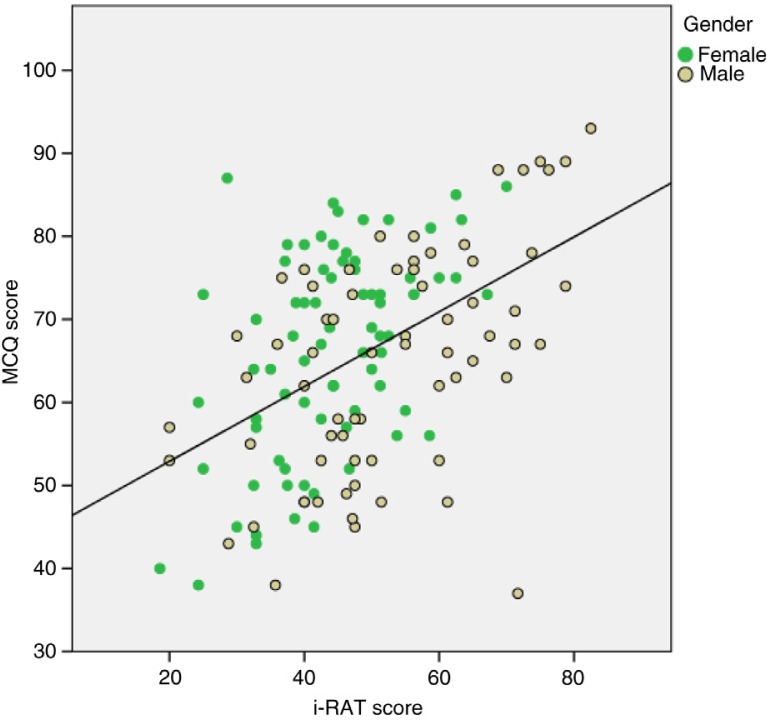
A moderate Pearson's correlation was found between student's i-RAT performance and their summative exam MCQ scores.

A weak correlation was seen between the students who attended the TBL sessions, their summative exam scores and their performance in the MCQs as shown in Fig. 3. A moderate Pearson correlation was found between student's i-RAT performance and their summative exam MCQ scores (*r*=0.47, *p*<0.001, *N*=143). Separate correlations for males and females were very similar (*r*=0.53, *p*<0.001, *N*=68 and *r*=0.50, *p*<0.001, *N*=75) as shown in Fig. 4.

We compared the summative assessment of the class for this block with the preceding block where TBL was not implemented. We found that there was an increase in the number of students achieving higher grades (Grade B and above) in this block when compared to the previous block (51.7% vs. 25%). The number of students at risk for lower grades (Grade B- and below) decreased in this block when compared with the previous block (30.6% vs. 55%).

### Student perceptions regarding the process of TBL

The results of online survey showed that 77% of the students agreed that the TBL helped in increasing the understanding of the course material. Approximately, 65% of the students were in agreement that i-RAT was a useful learning activity. Around 73% of the total students perceived the discussion of neurological scenarios in TBL as beneficial and they preferred this strategy over the didactic teaching strategy like lectures. In response to the question whether problem solving in groups was an effective way to learn neurosciences, 64% students were in agreement. Approximately, 80% of the students felt that the TBL helped them in better-preparing for the summative examinations. Around 77% of the students strongly agreed to TBL process helping them in information synthesising skills.

Student responses on communication and interpersonal skills during TBL sessions demonstrated 71% approval for problem solving and 68% for having a positive attitude for working with peers. Approximately, 44% of the student agreed that TBL helped in boosting confidence and overall improvement in communication skills.

## Discussion

We conducted this study because we wanted to explore the utility of TBL as an instructional strategy especially for a difficult subject such as Neurosciences at undergraduate level. We wanted to evaluate the effect of TBL on student learning and to analyse whether it has any effect on students’ performance in the summative assessment. This module was chosen for this intervention because of the perceived difficulty of the Neuroscience subject. To our knowledge, this is the first time a TBL strategy was implemented in any of the medical colleges of Saudi Arabia, utilizing state-of-the art teaching equipment, truly representing the first example of a novel teaching system within the region.

In our study, the mean t-RAT was significantly (*p*<0.001) better than the i-RAT across all groups and this is consistent with other studies (11, 12). Collectively, the mean lowest team score was higher than the mean i-RAT score. This finding is also in agreement with other studies (13–16). Our results indicate that students benefit from group discussion and their understanding and performance improve in the t-RATs.

In individual tests, the students scored an average of 47.48% indicating that the difficulty index of the questions was sufficient to stimulate discussion. Zgheib et al. recommend a difficulty level of 30–70% to improve group discussion and class performance in TBL (17). A weak Pearson correlation was found between student's i-RAT performance and their summative exam MCQ score. It has been shown by other studies that i-RAT is a good predictor of examination performance (14–18).

TBL provides larger benefit to low achievers compared with higher achievers (15). This study also demonstrated a weak positive impact on student's summative grades by implementing TBL. We found that the male students were better prepared for the TBL sessions than female students. This is a unique finding in our study as the system of teaching in our institute is gender segregated.

The male students’ performance in the TBL had a more favorable effect on their grades in the summative examinations as compared to female students. Similar findings have been reported by Weiner et al. in their study (14). The summative assessment showed an overall improvement in students achieving higher grades and decreased the number of students at risk for lower grades when compared to the preceding block however no statistical significance could be demonstrated. The same kind of trend has been mentioned in our previous study (12). A comparison was made between the same cohort of students over two consecutive blocks. However, such a comparison could not be made across different cohorts of students, since many variables other than TBL could explain any difference observed. Not only do different cohorts differ (some years are better than others), but detailed differences in how the two blocks were run (e.g. faculty teaching, organization, duration, assessment) could explain a difference. Such confounding variables would invalidate any statistical inference concerning the effect of the TBL.

### Student perceptions about TBL

57% of the students agreed that the content covered in the TBL helped them increase their understanding of the course material and led them to focus on the core information, 18.6% disagreed and 24% were neutral. Similar findings have been reported by Nieder et al. (15). Approximately, 45% of the students felt that they were well prepared for the i-RAT and that it was a useful learning activity. Approximately, 66% of the students felt that TBL process helped them in synthesising information, understanding the basic concepts of neuroscience and bridging the gap in knowledge through t-RAT. This is in agreement with findings reported by Nieder et al. (15). The same percentage of the students gave a positive feedback that the TBL activity helped them in exam-preparation, and they preferred TBL small group discussions to the didactic teaching sessions. Approximately, 67% of the students liked their experience in the small groups which helped them in improving their interpersonal and communication skills. This finding is also corroborated by other studies (14–18).

TBL is a tool catering to the needs of the rapidly changing practice of medicine and facilitates interprofessional and team-oriented learning (5). Parmelee et al. have emphasised the importance of TBL in fostering problem-solving and collaborative learning in a feedback-rich small group learning environment (5). In this study we also found that TBL promoted collaborative learning. Our results indicate that due to instantaneous feedback provided during TBL, students are never in doubt of the content covered in the sessions. A combination of small and large group dynamics leads to a high degree of interaction among learners leading to ownership and enthusiasm (6).

### Limitations of the study

In this neurosciences block the structure, function and pathogenesis was incorporated in one single block. We tried to compare our results with a historical control but due to confounding factors such as change in faculty and change in the block objectives from previous years, we were unable to do so. This is a major limitation of the study.

## Conclusion

This study showed that the students groups outperformed individuals in TBL. Male students were generally better prepared for the TBL sessions than the females. Moreover, TBL improved results for both low achievers and higher achievers. Team-based learning was received favorably by the students and their feedback showed that the content covered in the TBL improved their understanding of the course objectives and also helped in exam preparation. Students preferred TBL sessions to didactic sessions. We conclude that implementing a TBL strategy increased student responsibility for self-learning, and helped students to tackle ‘neurophobia’ in a difficult neurosciences block evidenced by their improved performance in the summative assessment.
